# An experimental analysis of body checking

**DOI:** 10.1016/j.brat.2006.01.015

**Published:** 2007-01

**Authors:** Roz Shafran, Michelle Lee, Elizabeth Payne, Christopher G. Fairburn

**Affiliations:** Department of Psychiatry, Warneford Hospital, Oxford University, Oxford OX3 7JX, UK

**Keywords:** Body checking, Eating disorders, Experiment, Body dissatisfaction, Body size estimation

## Abstract

The relationship between repeated body checking and its impact on body size estimation and body dissatisfaction is of interest for two reasons. First, it has importance in theoretical accounts of the maintenance of eating disorders and, second, body checking is targeted in cognitive-behavioural treatment. The aim of this study was to determine the impact of manipulating body checking on body size estimation and body dissatisfaction. Sixty women were randomly assigned either to repeatedly scrutinize their bodies in a critical way in the mirror (“high body checking”) or to refrain from body checking but to examine the whole of their bodies in a neutral way (“low body checking”). Body dissatisfaction, feelings of fatness and the strength of a particular self-critical thought increased immediately after the manipulation among those in the high body checking condition. Feelings of fatness decreased among those in the low body checking condition. These changes were short-lived. The manipulation did not effect estimations of body size or the discrepancy between estimations of body size and desired body size. The implications of these findings for understanding the influence of body checking on the maintenance of body dissatisfaction are considered.

## Introduction

The repeated critical scrutiny of one's body size, shape and weight is a characteristic feature of patients with eating disorders. This behaviour has been incorporated into descriptions of eating disorders (American Psychiatric Association, 1994; Fairburn & Harrison, 2003). Examples of such behaviour include examining oneself in the mirror, using the fit of clothes to judge whether size has changed, feeling for bones, seeking reassurance about shape, and making negative comparisons with others. The leading evidence-based treatment for bulimia nervosa addresses the frequent checking of body weight and shape (Fairburn, Marcus, & Wilson, 1993) and treatments for body image disturbance include a component designed to reduce such body checking and avoidance via exposure (Rosen, 1997).

Recent studies indicate that such body checking is normative, particularly among young women, but that people who have significant shape concern use the mirror to scrutinize the parts of their body they dislike more than those whose level of shape concern is low (Farrell, Shafran, & Fairburn, 2004). It has also been found that patients with eating disorders engage in more frequent body checking than normal controls and that the great majority of patients engage in such behaviour (Reas, Whisenhunt, Netemeyer, & Williamson, 2002; Shafran, Fairburn, Robinson, & Lask, 2004). Patients with obesity also engage in body checking (Grilo et al., 2005; Reas, Grilo, Masheb, & Wilson, 2005).

A consistent finding is that patients with eating disorders and those concerned about their shape scrutinize their disliked body parts more than those who are not as dissatisfied with their bodies and do not have an eating disorder (Reas et al., 2002; Shafran et al., 2004). What is not clear, however, is the exact nature of the relationship between body checking and body dissatisfaction. It is important to understand this relationship in order to understand the role of body checking in eating disorder psychopathology. One early suggestion was that “the frequent but brief checking of shape, while in a state of high arousal, magnifies perceived bodily imperfections’ (Fairburn, Shafran, & Cooper, 1999). Implicit in this hypothesis was that such behaviour may contribute to the overestimation of body size seen in some patients with eating disorders (e.g., Cash & Brown, 1987; Farrell, Shafran, & Fairburn, 2003). Later, it was suggested that body checking is an expression of the characteristic overevaluation of shape and weight in patients with eating disorders and that such behaviour “is likely to intensify their concerns” (Fairburn, Cooper, & Shafran, 2003, p. 514). Some support for this suggestion comes from positive association observed between body checking (and avoidance) and the overevaluation of weight and shape (Grilo et al., 2005; Reas et al., 2005; Shafran et al., 2004). Williamson's integrated cognitive-behavioural model of eating disorders (Williamson, White, York-Crowe, & Stewart, 2004) also highlights the importance of body checking, suggesting it may result from cognitive biases and/or binge eating, and lead to overconcern with body shape and size as well as fear of fatness.

The aim of this study was to manipulate body checking in a sample of women *without* eating disorders and to determine the immediate impact of this manipulation on body dissatisfaction and body size estimation. The second aim was to determine the duration of any impact of the manipulation. It was hypothesized that repeated checking of shape would magnify perceived bodily imperfections and would intensify concerns about shape. Although such a hypothesis may be best tested in patients with eating disorders, the theoretical accounts of Fairburn et al. (2003) and Williamson et al. (2004) suggest that these relationships are not intrinsically abnormal and should also occur in people without significant shape concerns. In addition, conducting such experimental manipulations in women with clinical eating disorders would be of ethical concern since it would involve intensifying potentially pathological behaviour. It was for these reasons we decided to investigate the relationship between body checking and body dissatisfaction in a non-clinical group of female participants.

## Method

### Participants

The patients comprised 60 women (mean age=24.72, SD=5.74) who had responded to advertisements asking for volunteers to take part in research on body image. Exclusion criteria were age below 18 or over 45, a current or previous history of an eating disorder, and current depression or suicidality (total score on the Beck Depression Inventory-II above 30, or 2 or above on the item assessing suicidal tendencies).

### Questionnaires/assessments

#### Eating disorder examination—questionnaire version (EDE-Q; Fairburn & Beglin, 1994)

This self-report questionnaire is based on the eating disorder examination (EDE; Cooper & Fairburn, 1987) Like the interview, it focuses on the participant's state over the preceding 28 days. It comprises 36 items that focus on the main behavioural features of eating disorders and those items needed to generate the EDE-Q subscales of Restraint, Eating Concern, Shape Concern and Weight Concern. It uses a seven-point forced-choice rating scheme for these subscales. Frequencies of key eating disorder behaviours are measured in terms of the number of days on which each particular form of behaviour occurred. The questionnaire has good reliability and validity (e.g., Reas, Grilo, & Masheb, 2006) and takes approximately 15 min to complete. At the end of this questionnaire, participants are asked to report their weight and height.

#### Body checking and avoidance questionnaire (BCAQ; Shafran et al., 2004)

This is a 23-item self-report questionnaire designed to assess checking and avoidance of body shape and weight. It uses a six-point forced rating scale which reflects the frequency with which each behaviour has been carried out in the previous four weeks. Scoring is as follows: 0=‘not at all/not interested’, 1=‘checked less than once a week’, 2=‘checked 1–6 times a week’, 3=‘checked 1–2 times a day’, 4=checked 3 or more times a day and 5=‘avoided doing so because of possible distress’. Examples of items include ‘pinching your thighs’ and ‘using the fit of your clothes to judge your body size’. Scores represent a mean across all items. The scale has good internal consistency and construct validity (Shafran et al., 2004).

#### Eating disorder inventory–body dissatisfaction subscale (EDI–DBS; Garner, Olmsted, & Polivy, 1983)

This nine-item subscale records individuals’ dissatisfaction with five body part (e.g. hips and thighs). Five items are positively phrased and are reverse-scored (e.g. ‘I think that my thighs are just the right size’).

#### Beck depression inventory II (BDI-II; Beck, Steer, & Brown, 1996)

This is a 21-item self-report instrument for measuring the severity of depression. The second version was used. This was developed to assess symptoms corresponding to the diagnostic criteria for depressive disorder specified in DSM-IV (American Psychiatric Association, 1994). The BDI-II measures the cognitive, behavioural and somatic severity of depression in adults and adolescents aged 13 and over. Each item is scored on a four-point scale ranging from 0 to 3, and the total score is obtained by summing the ratings for each item. Its reliability and validity are well established (Beck et al., 1996).

##### Visual analogue scales

Six visual analogue scales (VASs) were constructed for the study, two assessed the success of the experimental manipulation, and the others measured body dissatisfaction. The experimental manipulation scales asked participants to indicate how much of the previous 30 min had been spent ‘…scrutinising your body in detail, checking and looking for flaws’ (from 0=‘none of the time’ to 100=‘all of the time’). They were also asked to indicate how much of the previous 30 min had been spent ‘….standing back and seeing your body in a neutral way’ (from 0=‘none of the time’ to 100=‘all of the time’). These were completed after the 30-min intervention. The body dissatisfaction VASs were completed whilst participants looked at themselves in the mirror. They were asked to indicate ‘At this moment in time:’, ‘How concerned are you about your body shape?’ (0=not at all concerned, 100=extremely concerned), ‘How dissatisfied are you with your body?’ (0=not at all dissatisfied, 100=extremely dissatisfied), ‘On an emotional levels how ‘fat’ do you feel?’ (0=not at all ‘fat’, 100=extremely ‘fat’). Participants were also asked to identify the strongest thought that came to mind, and to rate ‘How much does this thought bother you?’ (0=not at all, 100=extremely). VASs have been shown to have good reliability and validity in a range of settings (McCormack, Horne, & Sheather, 1988).

#### Body Size Estimation task (BSE; see Shafran & Fairburn, 2002 and Farrell et al., 2003 for further information)

This task involves asking participants to adjust a “mirror-size” projected digital image whilst looking in an adjacent mirror to (a) match what they actually see in the mirror (‘actual’) and (b) how they would “like to look” in the mirror (‘desired’). BSE scores are calculated as a percentage of participants’ actual mirror image size, therefore a score of 110 would indicate that a participant had overestimated her size by 10%, whereas a desired size score of 90 would indicate that the desired body size was 10% less than her actual body size. Performance in this task is stable over a one-week period in healthy control women (Farrell et al., 2003). Using this task, patients with eating disorders significantly overestimate their body size relative to healthy controls, and BSE scores correlate significantly with eating disorder psychopathology (Shafran & Fairburn, 2002).

### Procedure

Participants completed the four questionnaires in the 24 h prior to attending the experimental session. Upon arrival, questionnaires were checked and participants were asked if they had ever had an eating disorder or had treatment for an eating disorder. Participants were excluded if there was previous history of an eating disorder, if they had a high BDI-II score (30+) or if they reported any objective bulimic episodes, compensatory behaviour or significant restriction (i.e., 4 or above on restraint items) on the EDE-Q. Assessments and manipulations were carried out by different experimenters in different rooms. Participants were asked to undress to their underwear, and to complete the body dissatisfaction VAS whilst looking in the mirror, followed by the body size estimation task. This involved manipulating the mirror-sized image four times to indicate an ‘actual’ body size and four times to indicate a ‘desired’ body size. The order of the manipulation was counterbalanced and the size of the image before manipulation was randomised. Participants were then asked to dress and were taken to another room to complete one or other experimental manipulation. Participants were assigned to the manipulation (high or low body checking) at random using random number tables. Participants were undressed to their underwear and looked in a full-length mirror in both conditions.

#### High body checking condition

Participants were asked to focus their attention specifically on the parts of their body with which they were dissatisfied. They were asked to examine and check these areas in specific ways to get more information about them. For example, they were asked to scrutinize parts of their bodies in the mirror, (e.g., chest, tops of arms, stomach, hips, bottom, thighs, calves) by standing at different angles, and seeing how much these areas protruded. They were asked to touch, feel, grab and wobble their flesh as they were looking at it in the mirror and describe how this made them feel, and to sit down on a chair and examine the spread of their thighs as they did so.

#### Low body checking condition

Participants were asked to look at all parts of their body in a mirror, each for a few seconds, starting from the head (e.g. hair, forehead, eyebrows eyes) and working down to the feet (e.g. calves, ankles, feet, toes). The experimenter called out the body parts. They were asked to describe each part in a neutral fashion. They were advised not to use either positive or negative language, but simply to describe what they saw as if they were looking at ‘someone else’. This technique was developed by Tuschen-Caffier and colleagues (e.g., Tuschen-Caffier, Vogele, Bracht, & Hilbert, 2003) and Wilson (e.g. Delinsky & Wilson, 2004) to help participants describe their body parts precisely and de-emphasize negative evaluations. If positive or negative language was used, participants were reminded of the original instructions to remain neutral throughout.

  After the manipulation had been completed, participants were asked to dress and return to the assessment room. They were then asked to complete the VAS manipulation checks. Once undressed again to their underwear, they were asked to look in the mirror and complete the four VAS scales assessing body dissatisfaction and to complete the BSE task once more in the same way as before.

After this second assessment, participants were dressed and moved to a third (neutral) room for a period of 30 min after which they returned to the assessment room for the third and final assessment (VAS for manipulation check, VAS for body dissatisfaction in their underwear looking in mirror and BSE in underwear).

#### Data analysis

2 (condition; high versus low body checking)×2 (time; before versus immediately after manipulation) mixed ANOVAs were used to analyse the data.

## Results

### Participant characteristics

The mean age, body mass index (BMI) and questionnaire scores for the 31 participants randomly assigned to the high body checking condition and the 29 assigned to the low body checking condition are shown in Table 1. The participants in the two conditions did not differ from each other on any of these measures prior to the experimental manipulation (all p>.05).

### Manipulation check

Independent sample *t*-tests indicated that the manipulation was successful in that those in the high body checking condition reported spending significantly more time during the manipulation “scrutinizing their body in detail, checking for and looking for flaws” than those assigned to low body checking condition (75% of the time as compared with 20% of the time, respectively; t(35)=6.20, p<.001), whereas those in the low body checking condition spent significantly more time during the manipulation “standing back and seeing their body in a neutral way” than those assigned to high body checking (85% of the time as compared with 25% of the time, respectively; t(35)=8.22, p<.001).

### Impact of the manipulation on the BSE task

The relevant means (and standard deviations) are presented in Table 2. For estimations of *actual* body size, there were no main effects of time (F(1,58)=1.73, p>.05ns) or condition (F(1,58)=0.03, p>.05ns), and no significant time by condition interaction (F(1,58)=0.03, p>.05ns). For *desired* body size, there were no main effects of either time (F(1,58)=0.02, p>.05ns) or condition (F(1,58)=0.05, p>.05ns) and no time by condition interaction (F(1,58)=0.17, p>.05ns). These findings indicate that the manipulation had no significant effect on estimations of actual and desired body size.

### Impact of the manipulation on attitudes towards body shape and dissatisfaction

The mean scores on four body dissatisfaction VASs are reported in Table 2. For item 1 (How concerned are you about your body shape?), there was a significant time by condition interaction (F(1,58)=5.68, p<.05). Further investigation via paired samples *t*-tests indicated that concern was significantly reduced in the low checking condition (t(28)=2.0, p=.05) but did not change in the high checking condition (t(30)=1.4, p>.05). For item 2 (How dissatisfied are you with your body?), there was a significant time by condition interaction (F(1,58)=11.21, p<.001), and paired samples *t*-tests indicated that after the intervention, dissatisfaction had increased in the high checking condition (t(30)=4.39, p<.001) but had remained unchanged in the low checking condition (t(28)=1.02, p>.05). The same pattern of findings was obtained for items 3 (How ‘fat’ do you feel?) and 4 (‘How much are you bothered by your strongest thought?’) (F(1,58)=11.72, p<.05; F(1,58)=5./55, p<.05, respectively) with an increase in the high checking condition (t(30)=3.91, p<.001 and t(30)=2.77, p<.05, respectively) and no change in the low checking condition (t(28)=1.27, p>.05; t(28)=.72, p>.05)). For item 4 (How much are you bothered by your strongest thought?) there was a significant time by condition interaction (F(1,58)=5.55, p<.05). The extent to which participants were bothered by their negative thought increased in the high checking condition (t(30)=2.77, p<.05) and remained stable in the low checking condition (t(28)=0.72, p>.05).

### Nature of thoughts when looking in the mirror

Participants were asked to write down the strongest thought they had when looking at themselves in the mirror. Of the 60 participants, 25 gave a shape/weight related thought both immediately prior to and after the intervention (14 in the high body checking condition and 11 in the low body checking condition). The other 35 participants expressed thoughts that were not specifically related to body shape e.g., ‘I wonder what the experimenter is thinking’, ‘I should get around to waxing my thighs’. The nature of each body-related thought in the 25 participants who expressed them was examined and rated by ML and EP (blind to condition allocation) on a scale from −2 (very negative) to +2 (very positive) where a rating of zero was neutral. Inter-rater reliability was very high (96% agreement, that is 48/50 of the ratings made). For the remaining two items, ratings differed by one point. Examples of thoughts and their corresponding ratings are as follows; −2: “I had a lot more loose fat than I thought”, −1: “slightly larger thighs than I’d like”, “0: My stomach is convex”, +1) “I have more definition on my waist than I thought”, and +2: “I’m really happy with what I see”. The relevant means are presented in Table 2. There was a significant time by condition interaction (F(1,35)=5.74, p<.05) with a trend for valence ratings to become more negative for those in the high body checking condition after the manipulation (t(20)=1.86, p=.07). No change in valence was found among those in the low body checking condition (t(15)=1.6, p>.05).

### Relationships amongst the variables and impact of variables to the response to manipulation

Correlation coefficients among indices of body dissatisfaction, body checking and body size estimation are presented in Table 3. Of particular note, the VAS used to assess body dissatisfaction was strongly associated with other indices of dissatisfaction (including the EDI subscale) and body checking.

Regression analyses were conducted to determine if baseline levels of dissatisfaction (assessed using the VAS, EDI dissatisfaction subscale and Shape and Weight concern subscale of EDE), body checking or body size estimation predicted the change in accuracy of size estimation. Results indicated that only initial accuracy in the body size estimation task was associated predicted change in accuracy after the intervention (r=-.28, p<.001). The same analyses were conducted for change in body dissatisfaction scores. Results indicated that none of these initial variables predicted change in dissatisfaction with the intervention (all *p*'s>.05).

### Duration of effects

The second aim of the study was to determine the duration of any impact of the manipulation. Scores obtained 30 min after the intervention are presented in Table 2. A series of paired *t*-tests with appropriate correction indicated that 30 min after the intervention, scores on all measures were comparable to scores before the intervention (all *t*'s<2, all *p*'s>.05) with the exception of dissatisfaction scores in the high checking condition, where scores were actually significantly lower 30 min after the intervention than prior to it (t(30)=2.24, p<.05)

## Discussion

This study aimed to examine the impact of manipulating body checking on body size estimation and body dissatisfaction in women without clinical eating disorders. The experimental manipulation was successful in that those assigned to the high body checking condition reported spending more time checking their body shape than those assigned to the low body checking condition who conversely spent more time examining their body in a neutral manner. The results indicated that those in the high body checking condition experienced a temporary, possibly contemporaneous, increase in body dissatisfaction, feelings of fatness and an increase in the strength of body-related self-critical thinking. These findings support the theoretical accounts of the relationship between body checking and shape concern and previous findings that suggest that body checking contributes to the maintenance of shape concerns.

Accordingly, they also suggest that such behaviour should be tackled in treatment. However, it is not surprising given that this is a non-clinical sample, that the low body checking condition had relatively little impact (only the variable ‘feelings of fatness’ were affected). Given that the intervention has positive findings in clinical samples (Delinsky & Wilson, 2004), it seems that the intervention is effective only when shape concerns are elevated.

The short duration of the effect of body checking is of interest both theoretically and clinically. From this study, it appears that the process of body checking only influences body dissatisfaction for a brief period and then body dissatisfaction returns to its baseline level. The findings are consistent with clinical observation that body dissatisfaction, feelings of fatness and self-critical thoughts fluctuate in the day. It is possible that these fluctuations are secondary to body checking. If this is the case, treatment that reduces the frequency of body checking is likely to reduce fluctuations in body dissatisfaction, feelings of fatness and self-critical thoughts, leaving the clinician able to address the other factors that are contributing to the patients’ concerns such as low mood, avoidance and, of course, the overevaluation of shape and weight (Fairburn et al., 2003).

The finding that body checking did not affect estimations of size is of interest. Given that numerous variables appear to influence size estimation (e.g., hunger level, specific instructions) and that size estimation is not stable (Cash, 2002; Smeets, 1997) it is of interest that body checking and the subsequent rise in feelings of dissatisfaction, do not seem play a part. It may be the case that body checking does not influence size estimation in those who are largely accurate in the first place. The vast majority of the present sample were not significantly overestimating their body size and although eating disorder groups as a whole score higher than non-clinical comparisons (Cash & Deagle, 1997), the group mean masks the fact that many individuals with eating disorders do not overestimate body size. Whether body checking does affect those who do overestimate their body size significantly needs to be determined. It is also of interest that body checking did not influence desired size. It may be the case that body checking draws attention to specific aspects of the body such as ‘wobble’ rather than overall size. Given the focus of attention on specific body parts during the checking procedure, this seems a plausible explanation but it warrants further exploration.

This study had a number of strengths. It is the first experimental analysis of the phenomena of interest, and the experimental manipulation was successful thus enabling a test of the hypotheses. The sample size was sufficient to test the hypotheses under investigation and state-of-the-art measures of body size estimation and desired body size were used. However, it also had a number of limitations. First, it was conducted on a non-clinical sample of women who did not have significant body dissatisfaction. The reason for this was primarily ethical and could be justified on the basis that the hypotheses from theories of the maintenance of anorexia nervosa specifically and eating disorders more generally (Fairburn et al., 1999, 2003) apply to non-clinical groups. However, one might speculate, based on previous research (Williamson et al., 2004), that the findings would be stronger in those with eating disorders or body dysphoria and possibly longer lasting. Such a pattern of findings could be attributable to both excessive concern and negative thoughts over body shape and studies that independently manipulated both body checking and negative thoughts about shape would be of interest. Examination of the impact of prolonged manipulations of body checking is also warranted and clinically relevant.

A second limitation of the current study is that VASs were used to measure some of the variables of interest. This was necessary given the brief time-frame over which the study was conducted but the psychometric properties of these scales were not established. Third, the sample did not allow the determination of whether body checking influenced a subset of participants (perhaps those who were overestimating their body size) although this is an area of interest. Finally, this study did not assess the overevaluation of shape and weight. This would also have been of interest given theories suggesting that body checking is an expression of this core psychopathology.

In conclusion, this is the first experimental analysis of the relationship between body checking and body dissatisfaction. It confirms the findings from psychometric studies demonstrating an association between these variables, and it also establishes a causal relationship. In addition, the experiment indicates that the effects are short-lived. Further exploration of the mechanism by which body checking influences body dissatisfaction would be of interest and clinical relevance.

## Figures and Tables

**Table 1:** Participant characteristics (means and standard deviations) pre manipulation^a^

	High body checking condition (n=31)	Low body checking condition (n=29)
Age	25.97 (6.79)	23.38 (4.04)
EDE-Q^b^ restraint	1.34 (1.08)	0.91 (0.80)
EDE-Q eating concern	0.65 (0.89)	0.51 (0.81)
EDE-Q shape concern	1.58 (1.17)	1.55 (1.28)
EDE-Q weight concern	1.13 (1.00)	1.14 (1.09)
BDI-II^c^	4.55 (3.93)	6.51 (4.45)
BMI^d^	22.41 (3.09)	23.10 (3.93)
BCAQ^e^	1.32 (0.57)	1.32 (0.93)
EDI–BDS^f^	34.48 (3.88)	33.39 (3.10)

a A series of independent *t*-tests indicated that there were no group differences prior to the experimental manipulation on any variable listed in Table 1 (df=58, all *t*'s<1.8, all *p*'s>.05).

b Eating disorder examination—questionnaire.

c Beck depression inventory-II.

d Body mass index.

e Body checking and avoidance questionnaire.

f Eating disorders inventory—body dissatisfaction subscale.

**Table 2:** Mean body size estimation scores (and standard deviations) pre and post manipulation

	Pre-manipulation		Post-manipulation (immediate)		Post-manipulation (30 min later)	
	High body checking	Low body checking	High body checking	Low body checking	High body checking	Low body checking
*BSE task*						
Actual size	108.82 (7.62)	109.00 (7.05)	107.54 (9.01)	108.02 (7.72)	106.98 (6.36)	107.96 (8.19)
Desired size	96.15 (9.84)	95.14 (10.44)	95.86 (10.24)	95.75 (13.13)	94.13 (7.33)	95.25 (10.57)
*VAS*						
Q1: How concerned are you about your body shape?	43.74 (24.55)	38.51 (22.04)	46.48 (24.14)	34.86 (20.94)	39.71 (25.31)	31.14 (22.58)
Q2: How dissatisfied are you with your body?	38.61 (22.27)	36.48 (22.35)	46.29 (21.40)	33.84 (21.80)	34.44 (22.67)	31.62 (22.95)
Q3: How ‘fat’ do you feel?	37.55 (23.25)	35.21 (24.57)	44.85 (23.86)	32.14 (21.30)	33.95 (22.29)	28.31 (22.29)
Q4: How much does the thought bother you?	37.45 (25.14)	34.66 (25.78)	45.68 (23.96)	32.16 (23.60)	30.55 (23.37)	28.41 (24.08)
Valence of thought	−0.64 (0.93)	−1.00 (1.09)	−1.50 (0.65)	−0.55 (1.04)	−0.36 (1.21)	−0.73 (1.19)

**Table 3:** Correlation coefficients between body size estimation, indices of body dissatisfaction and body checking

	BSE (‘desired’)^a^	BSE (‘actual’)^b^	BDI-II^c^	EDE-Q shape^d^	EDE-Q weight^e^	BCAQ^f^	EDI-BDS^g^	VAS (body dissatisfaction)^h^
BSE (‘desired’)	—	.30*	−.18	−.43**	−.40**	−.40**	.10	−.34**
BDI-II	—	—	—	.42**	.50**	.38**	.10	.29*
EDE-Q shape	—	—	—	—	.79**	.73**	.30*	.66**
EDE-Q weight	—	—	—	—	—	.64**	.37**	.52**
BCAQ	—	—	—	—	—	—	.30*	.47**
EDI–BDS	—	—	—	—	—	—	—	.27*

*N*=60; *p<.05; **p<.01.

a Body size estimation task—desired size.

b Body size estimation task—actual size.

c Beck depression inventory—second edition.

d Eating disorder examination—questionnaire version (shape concern).

e Eating disorder examination—questionnaire version (weight concern).

f Body checking and avoidance questionnaire.

g Eating disorders inventory—body dissatisfaction scale.

h Visual analogue scales (for body dissatisfaction).
